# Opportunities for Cost-Sharing in Conservation: Variation in Volunteering Effort across Protected Areas

**DOI:** 10.1371/journal.pone.0055395

**Published:** 2013-01-30

**Authors:** Paul R. Armsworth, Lisette Cantú-Salazar, Mark Parnell, Josephine E. Booth, Rob Stoneman, Zoe G. Davies

**Affiliations:** 1 Department of Animal and Plant Sciences, University of Sheffield, Sheffield, United Kingdom; 2 Department of Ecology and Evolutionary Biology, University of Tennessee, Knoxville, Tennessee, United States of America; 3 en:mapping GIS & Spatial Solutions, Sheffield, United Kingdom; 4 Yorkshire Wildlife Trust, York, United Kingdom; University of Otago, New Zealand

## Abstract

Efforts to expand protected area networks are limited by the costs of managing protected sites. Volunteers who donate labor to help manage protected areas can help defray these costs. However, volunteers may be willing to donate more labor to some protected areas than others. Understanding variation in volunteering effort would enable conservation organizations to account for volunteer labor in their strategic planning. We examined variation in volunteering effort across 59 small protected areas managed by Yorkshire Wildlife Trust, a regional conservation nonprofit in the United Kingdom. Three surveys of volunteering effort reveal consistent patterns of variation across protected areas. Using the most detailed of these sources, a survey of site managers, we estimate that volunteers provided 3200 days of labor per year across the 59 sites with a total value exceeding that of paid staff time spent managing the sites. The median percentage by which volunteer labor supplements management costs on the sites was 36%. Volunteering effort and paid management costs are positively correlated, after controlling for the effect of site area. We examined how well a range of characteristics of the protected areas and surrounding communities explain variation in volunteering effort. Protected areas that are larger have been protected for longer and that are located near to denser conurbations experience greater volunteering effort. Together these factors explain 38% of the observed variation in volunteering effort across protected areas.

## Introduction

The expansion of protected area networks is limited by costs of setting up and managing sites in line with conservation objectives. Management costs of protected areas can be substantial, potentially exceeding the cost of acquiring sites to begin with when funded on an endowment basis [1]. Of these management costs, paid staff time is the largest cost item on many protected areas. Conservation organizations can offset management costs by relying on volunteer labor for some aspects of protected area management. Volunteers are often involved in monitoring and research [2], [3], [4] habitat management [5], control of invasive species [6], and activities related to protected area establishment [7]. The cost-sharing contribution made by volunteers sometimes greatly exceeds actual expenditures on particular conservation activities [8], [9].

Conservation organizations factor opportunities for cost-sharing and leveraging external resources into their strategic planning, and this is now starting to be considered in theoretical conservation planning analyses [10], [11]. Volunteer labor provides one version of cost-sharing. However, like other forms of cost-sharing (e.g. partnerships with other conservation groups, donor support), volunteer labor is often subject to spatial constraints. Volunteers may be willing to provide more labor to protected areas that are near their homes or that they care more about for whatever reason. In order to plan how best to manage volunteers and to utilize the cost-sharing that they provide most effectively, conservation organizations need to understand what explains patterns of variation in volunteering effort.

Variation in the availability of volunteer labor to help manage protected areas may be influenced by characteristics of the protected area itself and by variation in the surrounding communities from which volunteers are drawn. Protected areas vary in all manner of characteristics (e.g. size, habitat characteristics, species composition, and management needs [12]), including how people interact with them (e.g. people’s knowledge of them and willingness to visit [13], [14]). Households vary in their willingness and motivations for charitable giving with income, age, education, environmental preferences and other factors [15], and typically have clumped geographic distributions when scored against such axes [16]. Variation in the distribution of households sharing common characteristics may lead to a greater availability of volunteering labor for protected areas near some human communities than others.

**Figure 1:**
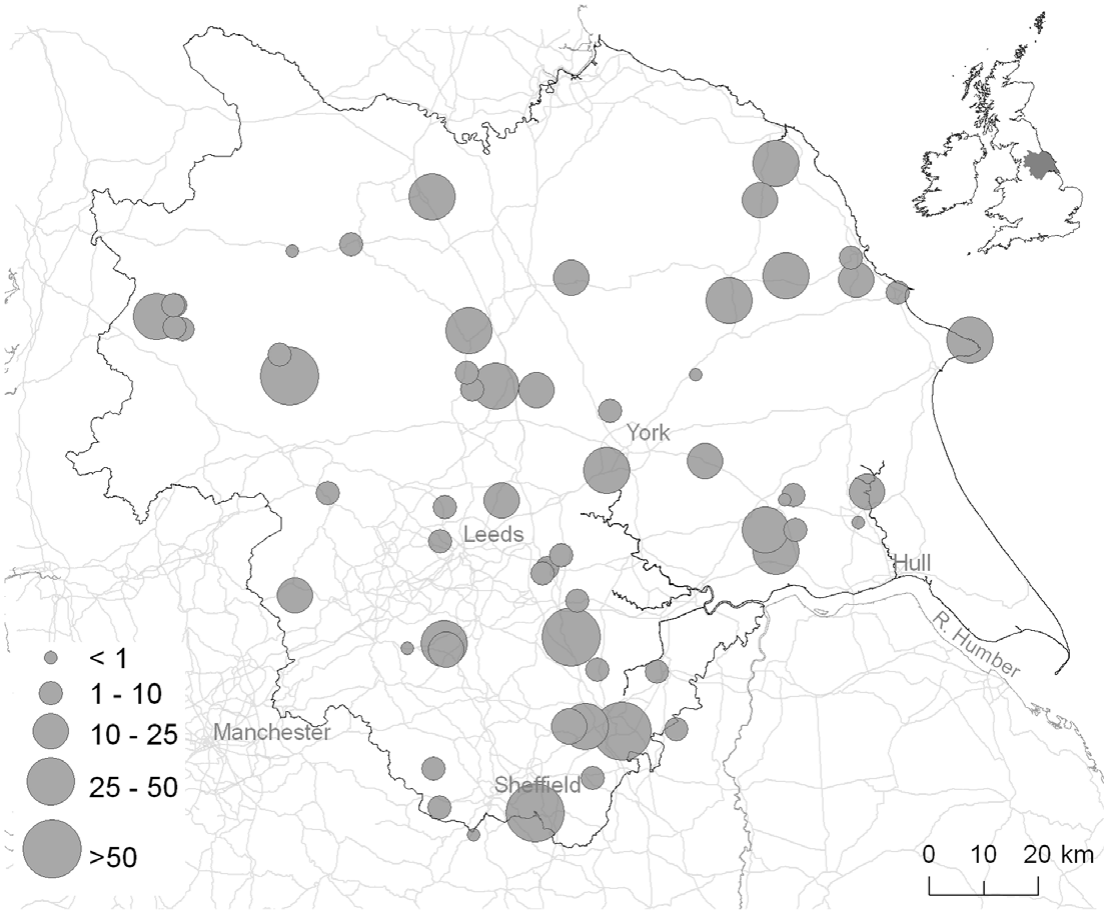
Yorkshire Wildlife Trust protected areas in Yorkshire, UK (inset). The size of circles provides an indication of site area on a categorical scale (<1, 1–10, 10–25, 25–50, >50 ha) that is only used for illustration purposes in this figure - all analyses treat site area as a continuous variable.

We examine variation in volunteering effort across a set of small protected areas in Yorkshire, UK (Fig. 1) that are managed by the Yorkshire Wildlife Trust (YWT). YWT is a regional conservation nonprofit that is organized similarly to local land trusts in the US and elsewhere. As part of their business model, YWT rely on volunteering effort for delivering elements of their overall conservation mission. For example, YWT volunteers undertake activities including monitoring biodiversity, controlling invasive plants, restoring habitats, administration and providing conservation GIS support. Like many land-trusts, YWT started with no paid staff but took on management of reserves anyway, meaning volunteer labor was essential. As the organization grew and employed paid reserves officers, YWT continued to value the cost-sharing contribution made by volunteers, but also began to recognize additional benefits from its conservation volunteering program. For example, participating in volunteering activities can foster a greater understanding of conservation issues among volunteers themselves [17], [18], potentially leading to further contributions to conservation by these individuals in the future.

We investigate how well site and surrounding community characteristics explain variation in volunteering effort across YWT’s protected areas. To explore the cost-sharing contribution from volunteers, we compared the spatial distribution and value of volunteering effort to the distribution of actual management costs on these sites. We leave considerations of broader benefits of volunteering for future work. Our focus on variation in volunteering effort across protected areas to inform conservation planning complements other studies on conservation volunteers that emphasize motivations for participation [19], [20], [21], potential well-being benefits individuals obtain from volunteering [22], [23], [24], and the reliability of the ecological data gathered from volunteer monitoring programs [25], [26].

## Materials and Methods

### Ethics Statement

Methods for collection of data, analyses of data and uses of data in this project were specifically approved by the University of Sheffield Research Ethics Committee. For each survey instrument, participants were first provided with a statement discussing the nature and purpose of the survey and use of the data should they choose to participate. For questionnaires that were mailed to volunteers, participants were free to choose whether to complete and return the survey, a form of consent approved by the University of Sheffield Research Ethics Committee. Questionnaires provided to site managers were administered in person with informed consent being provided verbally in advance over the phone and again at the start of the relevant interview. Participants were also encouraged to stop completing the questionnaire at any time should they so wish with completion of the questionnaire further signifying their consent. These forms of consent were approved by the University of Sheffield Research Ethics Committee for the site manager survey (in lieu of alternatives, such as written consent) in light of the data being collected and type of survey instrument (a survey of protected area management practices administered to employees of a registered charity that included no personal information of any kind).

### Volunteering Effort

We examine the distribution of volunteering effort across 59 small protected areas managed by YWT (Fig. 1). The protected areas vary in size by a factor of over 600 (median area = 7.9 ha, range = 0.3–154.9 ha), encompass a variety of habitat types, and differ in their proximity to cities and towns (Table 1). In autumn 2008, we conducted a closed-form, face-to-face questionnaire survey with 12 staff members active in protected area management. We asked each staff member to estimate the number of days of volunteer labor (defined as all unpaid labor) devoted to each protected area for which they had management responsibility (range = 1–17 protected areas per individual).

**Table 1:** Variation in volunteer effort and sample protected area characteristics.

	Q1	Median	Q3
Volunteer labor (person days/yr)	2	20	50
Site area (ha)	3.0	7.9	28.3
Year acquired	1981	1986	1996
Steepness of site (slope coefficient)	0.01	0.05	0.11
No. postcodes	875	2393	5755
Deprivation (IMD, %)	11.3	21.8	28.4
Outdoor recreation (%)	33	40	61
Management cost (2008 GBP£)	865	2191	4195

Median and lower and upper quartiles for site manager estimate of volunteer labor, predictor variables included in the multiple regression and overall management costs. All values are given per site (n = 59).

We compared variation in volunteering effort across protected areas estimated by this site manager survey with estimates from two additional sources as a check on data quality. First, we mailed a separate closed form questionnaire to all volunteers included on YWT’s volunteer mailing list (613 individuals) in September 2007 that asked them how much time they had spent volunteering on each protected area in the previous three months. 192 questionnaires were returned, a response rate of 31%. This postal questionnaire had the potential to provide information on all 59 sites, but yielded many zeros, likely some of which indicated non-responses. Variation in volunteer effort across protected areas estimated by the postal survey was correlated with that estimated by the site manager survey whether excluding (Spearman’s rho = 0.89, p<0.0001, n = 15) or including (Spearman's rho = 0.41, p<0.01, n = 59) sites that recorded a zero in the postal survey. We then compared spatial patterns of volunteering effort from the survey of site managers with YWT’s own records on volunteering on 20 protected areas. For these sites, YWT claimed volunteer labor as match funding in proposals for government grants (Heritage Lottery Fund) to fund conservation activities. Again, the estimates of volunteering effort on protected areas were positively correlated (Spearman’s rho = 0.76, p<0.0001, n = 20). Because our estimates of volunteering effort across protected areas are correlated when using three different sources, we can be more confident that our site manager survey is estimating actual spatial variation in volunteering effort. For our analyses of what explains variation in volunteering effort, the site manager survey has advantages over the other two data sources in that it offers more complete and consistent spatial coverage of YWT protected areas.

The volunteers in question vary greatly in their level of expertise, from those with detailed knowledge of particular sites or taxa to those with little training or experience. To convert site managers’ estimates of volunteer effort in days to an approximate economic value of volunteer labor, we assumed conservatively that volunteers put in on average 7 hours of labor per day and the value of that labor is 2008 GBP£7 per hour. This hourly wage rate for volunteers is greater than the UK minimum wage of £5.52 per hour at the time of our study and is around 55–60% of the value of paid staff active in reserve management when this is prorated to an hourly wage.

### Predictor Variables

We examined how well a range of characteristics of protected areas explained variation in volunteering effort (Table 1). Site area is a key determinant of management costs on these small protected areas [1]. To test for an effect of area on the cost-sharing contribution made by volunteers, we analyzed volunteer labor on a per site basis, including site area as a required covariate in all models.

To test for an effect of how long each site had been in conservation management with YWT, we included the year YWT acquired the site as a candidate predictor of volunteering effort.

We also examined whether habitat characteristics of sites affected volunteer effort. We classified sites by dominant habitat type using a 3 way categorical variable (woodland/shrub, grassland/marsh, other) that we included in the models using dummy variables, which could adjust the intercept value. We also examined whether people were less willing to volunteer on protected areas characterized by steeper, more difficult terrain.

Next we investigated how well characteristics of surrounding communities explained variation in volunteering effort. We identified surrounding communities based on a 15-minute travel time delimited catchment around each site, calculated using the UK Integrated Transport Network (road routing information), with the ESRI ArcGIS Network Analyst extension. To test for an effect of population density, we assessed the number of postcodes found within this area. A postcode contains 15.9 households on average. Our choice of a 15-minute car journey was admittedly arbitrary, but sensitivity testing revealed very similar patterns of population around protected areas when measured instead using 5 minute, 10 minute, 20 minute and 25 minute travel-time delimited catchments. We chose to use postcode number rather than more direct estimates of population or numbers of households from the UK census, because the number of postcodes correlates closely with household number, but is available a finer resolution than census data, something that is particularly important in more rural areas.

We also tested whether household characteristics in these surrounding communities influenced levels of volunteering effort. First, we examined whether levels of volunteering effort varied with levels of social deprivation in surrounding communities. As a measure of deprivation, we used the UK government’s Index of Multiple Deprivation (IMD). This index integrates seven distinct dimensions of deprivation (e.g. income deprivation, or health deprivation and disability) [27], several of which have been found to be associated with lower levels of volunteering in national surveys of overall involvement in volunteering [15]. We calculated a population density weighted average IMD score based on the overlap of the 15 minute travel-time delimited catchment with Lower Super Output Areas from the UK census, the finest grain over which the IMD is reported.

We also examined household preferences for outdoor recreation. People volunteering their time to charitable activities face many choices about the type of organization they wish to support and activities they wish to participate in. We hypothesized that, among households active in volunteering, those that also spend more leisure time on outdoor activities would be more likely to devote any volunteering labor to protected area management for an environmental charity than to some other charitable cause or activity. To construct an index of preferences for outdoor recreation, we relied on the English Leisure Visits Survey 2005, a stratified national survey of the leisure activities of UK households. We focused on responses of surveyed households that fell within each of the travel time delimited catchments. We calculated the proportion of leisure days spent by these households on the four outdoor leisure activities (walking, cycling, and visiting a beach or a park) from a list of 18 outdoor and non-outdoor leisure activities included in the survey.

YWT managed 78 sites at the time of the site manager survey. However, information on preferences of surrounding households for outdoor recreation were sparse for some of the most rural sites and we discarded any sites from the analysis where the English Leisure Visits Survey surveyed fewer than 5 households within our 15 minute travel time delimited catchment. This left a sample of 59 protected areas. We do not believe that proceeding with this reduced set of 59 sites poses problems for our design, because the sites that we include still spanned considerable variation in all predictor variables (Table 1).

### Expenditure on Site Management

We compare the value of volunteering effort to expenditures on site management by YWT. YWT provided financial details through audited accounts regarding direct expenditure on each site. We used the average of such expenditures between 2004 and 2008. Expenditures covered items such as habitat management and equipment maintenance as well as administrative costs (e.g. legal fees, meeting costs, printing) where these could be attributed to the management of a particular site. We also included an estimate of paid staff time allocated to each protected area. This estimate was obtained as part of the site manager survey. Site managers estimated the percentage of their time allocated to each site for which they had some management responsibility. We converted these values into a cost equivalent based on the salaried year and relevant staff member’s wage. We combined the two values (direct expenditure+paid staff time) into a single estimate for expenditures involved in managing each site faced by YWT. Further detail of these management cost data are given in Armsworth et al. [1]. Expenditures were per year and converted to 2008 GBP£ equivalent using the Consumer Price Index.

### Analyses

We used multiple regression with generalized linear models to examine variation in volunteering effort. We log transformed site area, mean steepness and our population density measure in all analyses. We did not consider interaction terms, having no a priori reason to focus on a particular subset of interactions from among the many that are possible. We tested predictor variables for collinearity and all tolerances were within acceptable levels.

We used a generalized linear model assuming a negative binomial error structure with log-link function. This particular error structure is appropriate because we analyze count data with potential clumping caused by some volunteering activities being undertaken by teams. We constructed all possible models given the set of predictor variables (64 models) and relied on AIC competition to identify a set of models that offer parsimonious explanations for variations in the data. We used AICc when ranking models to adjust for small sample sizes, and identified those having AICc values within 2 points of the minimum observed. Dispersion for models within this set, calculated as residual deviance divided by the degrees of freedom, was 1.27–1.29, indicating slight over-dispersion. We constructed a model average across this set of parsimonious models based on AIC weights. As an indicator of the explanatory power of the models, we report the explained deviance or pseudo r^2^ value (1-residual deviance/null deviance) [28].

We tested the residuals from the model average for evidence of spatial autocorrelation. We used SAM v4.0 to test the significance of Moran’s I across 10 equal distance classes between sites, testing significance using 200 randomizations. The residuals from the model average showed no evidence of spatial autocorrelation and we therefore used non-spatial models.

We also wanted to examine how variation in volunteering effort relates to expenditures on site management. Because both variables show a strong effect of area, we calculated partial correlations after controlling for area.

## Results

### Volunteering Effort

The survey of site managers estimated nearly 3200 days of volunteer labor per year were spent on the 59 sites (median of 20 days per site per year). The distribution of volunteering effort across sites was right-skewed. Some received no volunteer labor at all, whereas one (Potteric Carr) received 620 days of volunteer labor (19% of the total).

When we assume conservatively that volunteers put in on average 7 hours of labor per day and the value of that labor is £7 per hour, the estimated value of volunteer labor on these protected areas was £156 000. For comparison, YWT’s expenditure on managing these protected areas including paid staff time (worth £141 000) totaled £957 000 that year. As such, the cost-sharing contribution of volunteer labor was around 16% of overall site management costs, exceeding the value of paid staff time spent in managing sites. The median percentage by which volunteer labor supplemented overall management costs across the 59 sites was 36%.

### Variation in Volunteering Effort

When seeking to explain patterns of variation via multiple regression, four models had AICc values within two points of the minimum and offered parsimonious explanations of the data. In Table 2, the final column indicates the proportion of the variability explained by the models, with all four models having values of 0.38. Our measure of neighboring population density and the time the site has been in conservation management appear in all four models, along with the required covariate of site area. For each of the four models and the model average, the 95% confidence limits for the coefficients of these three predictor variables do not span zero. More volunteering effort is devoted to sites that are bigger, have larger population sizes nearby and that have been in conservation management for longer. The other variables contribute little to explaining variation in volunteering effort.

**Table 2:** Parsimonious set of models explaining variation in volunteering effort across sample protected areas.

Model	Intercept	Log site area	Year acquired	Dominant habitat	Log site steepness	Log postcode density	Deprivation (IMD)	Outdoors recreation (%)	AICc	Akaike Weight	Variation explained
1	59.78±28.71	0.67±0.12	−0.03±0.01	–	–	0.37±0.15	–	–	512.9	0.44	0.38
2	59.45±28.85	0.71±0.12	−0.03±0.01	–	–	0.54±0.21	−0.03±0.03	–	514.3	0.23	0.38
3	66.68±28.81	0.62±0.13	−0.03±0.01	–	−0.11±0.14	0.35±0.15	–	–	514.7	0.18	0.38
4	60.06±28.73	0.69±0.12	−0.03±0.01	–	–	0.4±0.16	–	0.005±0.01	515.0	0.16	0.38
**Model average**	61.0±28.89	0.67±0.13	−0.03±0.01	0.00±0.00	−0.02±0.05	0.41±0.18	−0.01±0.01	0.001±0.002			–

The four models having AICc values within 2 points of the minimum AICc value observed and the model average across this set. Columns show parameter estimates and standard errors, AICc values, model weights, and explained deviance or pseudo r^2^ for which the values are equal only to within rounding error reported in the Table.

### Covariation with Management Costs

Volunteer effort and the overall management cost of the sites are positively correlated even after controlling for site area, (partial Spearman’s rho = 0.35, p<0.01, n = 59).

## Discussion

Efforts to protect habitat through the establishment of protected areas are constrained by costs of managing these sites, something increasingly recognized in conservation planning studies [29], [30]. Volunteer labor can provide one source of cost-sharing in protected area management, potentially allowing more sites to be protected for a given budget. The availability of volunteer labor could also serve as an indicator of levels of support for protected areas from local communities. For these reasons, being able to predict variation in the availability of volunteer labor could help inform future conservation planning, both regarding annual budget allocation decisions among existing protected areas and potential future acquisitions, by allowing more accurate estimation of long-term management costs.

Conservation organizations and agencies active in protected area management vary widely in the business models that they follow and opportunities that they have available [31]. Obviously our results present a case study, albeit of an organization that follows a common, land trust-like business model and that, while slightly larger than average for a conservation nonprofit, is not exceptionally so. That being said, YWT tend to draw more on volunteer labor than do some of their peer organizations in the UK. It would be interesting to compare our findings to similar studies focused on other contexts and conservation organizations, including public agencies for which costs faced when establishing and managing protected areas, as well as opportunities for meeting those costs, can be quite different.

The overall cost-sharing contribution of volunteers on YWT’s protected areas was equivalent to a relatively modest 16% of overall site management costs. However, this figure is affected by the skewed distribution of volunteer labor and paid management costs. If we were to drop one site (Potteric Carr) that is something of an outlier for both the amount of volunteer labor donated and paid management costs [1], the overall contribution of volunteer labor is worth 54% of total management expenditure on the remaining 58 protected areas. On average across all 59 sites, the contribution of volunteer labor is worth 36% of the expenditure on management costs.

These cost-sharing figures should be considered ball-park estimates only. The actual cost-sharing contribution of volunteer labor would be under- or over-estimated if volunteers are more or less able to deliver on required tasks than paid staff than is estimated by the lower salary rate we used of £7 per hour, or if YWT would not have undertaken some of the same activities had volunteer labor not been available (a common problem to replacement cost estimation [32]). We have no estimate of the conservation outcomes (e.g. improvement in habitat condition of priority habitats across the protected area network) resulting from paid versus volunteer labor with which to make a more refined estimate. Even were such data available, additional benefits from a reliance on volunteers would be missed. In particular, we could not yet account for any future contributions individuals make to conservation that stem in part from experiences they gain when participating in volunteering [33]. Finally, we do not account for wider social benefits of volunteering, which could be substantial, and instead we focus only on the value of volunteering to YWT.

The main focus of our analysis is on explaining relative variation in volunteer labor across protected areas rather than providing an overall estimate of the combined value of this labor. With three simple descriptors of protected areas and their surrounding communities, we are able to explain around 38% of the variation in volunteer effort across sites. Protected areas that are larger, have been protected for longer and that are located near denser conurbations attract greater volunteer labor. The directions of these relationships align with expectation. However, much of the utility of such analyses derives from identifying what subset of relationships from among a plausible set that could be important actually prove important for explaining the observed variation. As such, the finding that, in contrast, household characteristics in the surrounding communities (as described by deprivation levels and preferences for outdoor recreation) and ecological characteristics of the protected areas (broad habitat type and elevation gradients) explained little of the observed variation in volunteering effort is itself a useful result.

Various take-homes for conservation groups are suggested by the subset of variables that were important for explaining variation in volunteering effort. For example, protected areas that have been established for longer attract more volunteer labor, perhaps because they become better known to or valued by their surrounding communities. This result highlights one way in which costs faced by conservation organizations in managing a protected area may change through time. Typically, estimates of variation in management costs have been based on snap-shot data [1], [34], [35], and detailed inter-temporal projections of management costs for different protected areas are not yet available, despite being needed to project future budget allocations and endowment needs. Other important take-homes arise from the increase in volunteering effort observed near denser conurbations. Conservation planning debates often focus on the relative priority that should be assigned to protecting areas near human habitation where land is threatened but also more expensive versus to more remote areas where larger areas can be purchased that are less threatened [36], [37], [38]. Rather than an either/or decision, the optimal strategy will likely involve protecting a portfolio of sites that spans this gradient and that is determined by the balance of marginal benefits and costs of different location choices. Our estimate of the increase in volunteer labor near more densely populated areas quantifies one relevant benefit of positioning protected areas closer to people.

An interesting question is whether volunteer labor actually substitutes for expenditure on protected area management. Our study design cannot answer this question directly, but it is interesting to note that volunteer labor and site management costs are positively correlated, even after controlling for the effect of site area. Were there a very strong substitution effect, one might expect a negative correlation here. A positive correlation may instead suggest that volunteer labor and paid staff time are partial complements. Indeed, a common experience of conservation groups is that managing volunteers makes demands on paid staff time [5]. For 20 of the study protected areas, the potential for management costs and volunteer labor to be complements may be accentuated, because YWT claimed the value of volunteer labor on these protected areas as a match-funding contribution on proposals for government grants. This situation raises a broader question of the degree to which YWT were directing volunteering effort and our analysis is revealing the organization’s management strategy versus the extent to which our analysis reveals the preferences of volunteers themselves. The answer likely lies somewhere in between. There is some coordination of volunteer activity (e.g. organized “blitz” days on particular sites), but at the same time, much volunteer effort on these sites is entirely bottom-up. Moreover, some of the volunteer labor is not fungible in space. Some volunteer groups are tied to particular protected areas (e.g. Friends of Potteric Carr) and many volunteers will only give time or will give more time to helping on protected areas near their homes. Finally the factors that emerged as important for explaining variation in volunteering effort also suggest an important bottom-up component. YWT did not focus their own management efforts during this period towards sites that had been protected for longer or were near more densely populated areas [1], despite these being the places that received the most volunteering effort.
